# Quantifying the consequences of unsustainable sand mining and cascade dams on aspects in a tropical river basin

**DOI:** 10.1038/s41598-024-51405-z

**Published:** 2024-01-12

**Authors:** Binh Quang Nguyen, Sameh A. Kantoush, Tetsuya Sumi

**Affiliations:** 1 Water Resource Center, Disaster Prevention Research Institute (DPRI), Kyoto University, Kyoto, 611-0011 Japan; 2 The University of Danang-University of Science and Technology, 54 Nguyen Luong Bang, Da Nang, Vietnam

**Keywords:** Hydrology, Natural hazards

## Abstract

Human interventions at the river basin scale, such as sand mining and hydropower dam construction, have profoundly affected hydrological and hydraulic alteration regimes, sediment budgets, and morphological changes worldwide. Quantifying the consequences of unsustainable ongoing sand mining and hydropower is crucial for obtaining sediment load data and managing hydrogeomorphology. In this study, comprehensive long-term consecutive four-field monitoring, statistical methods, and hydrological models (SWAT) were applied to quantify the spatiotemporal changes in long-term discharge and sediment load from 1996 to 2020 for the tropical river of the Vu Gia Thu Bon (VGTB) in the central region of Vietnam. The SWAT model was calibrated from 1996 to 2010, validated from 2011 to 2020 and showed good performance for daily discharge and monthly sediment. The evolution of river bathymetric data (2010, 2015, 2018, and 2021) was analysed to clarify the upstream sediment supply trapped in the riverbed and how the sand mining volume was removed. The results showed that the mean annual sediment in the Vu Gia and Thu Bon Rivers decreased by 57.3% and 23.8%, respectively, in the postdam period compared with the predam period. The thalweg elevation decreased at the Ai Nghia and Giao Thuy stations from 2010 to 2021 by 1.8 m and 3.9 m, respectively. The water level decreased by 21.1% at Ai Nghia and 44.3% at Giao Thuy. Dam development, sand mining, and changes in land use are the main factors responsible for flow discharge and sediment morphodynamic alterations. Morphological change have increased the water transfer rate from the Vu Gia River to the Thu Bon River through the Quang Hue channel. Downstream of the Vu Gia River, water transfer and riverbed incision have decreased flow discharge and water level and increased saltwater intrusion in recent years. As a result, water shortages induced by saltwater intrusion during drought periods have emerged as a significant constraint in hindering the domestic water supply and agricultural production.

## Introduction

The flow regime and its components influence downstream deltaic systems worldwide^1^. The duration and intensity of flow discharge are fundamentally dependent on climatic variables and the operation of basin-wide infrastructures such as hydropower dams and water diversion structures^2^. Sediment transport is critical for morphological changes and impacts river hydrology cycles^3,4^. River sediment is much more connected to the activities of humanity and other species. Therefore, managing river sediment is one of the most important considerations for ensuring the maximum benefits of rivers. The evidence from many rivers worldwide indicates that sediment load has been substantially disturbed due to dam construction, sand mining, land use-land cover (LULC) change, and climate variability^5–8^. Recent studies also show that these anthropogenic and climate-induced changes can significantly alter hydrology, ecological health, and water resources at very large scales^9–11^. Understanding the morphological changes and sediment budget is crucial for determining the flow capacity to reduce flood risks, sediment erosion, and deposition from mountainous areas to the sea. Therefore, assessing the impact of human activities on sediment loads can provide scientific insight into the sediment budget and complex basin hydrology and aid in the development of strategies for river basin management and sustainability. Until now, there has been significant progress in river sediment research from experiments to actual, specific methods to integrated approaches; from local problems to global issues, and from academic perspectives to policy implications^12^.

A boom in reservoir construction has occurred mainly in regions with emerging economies for irrigation, drinking water, hydropower generation, and hydrological hazard control. It is undeniable that reservoirs are necessary for flood control and securing water supplies for agriculture and the environment in tropical river basins with complex climate characteristics^13^. However, despite their benefits, reservoirs remain controversial owing to their potentially negative impacts on discharge, sediment, and morphology^14^. Dams trap substantial amounts of sediment, reducing the sediment supply downstream^7,15–17^. A sediment deficit may occur below a dam, depending on the relative sediment supply and transport capacity change. Nevertheless, most commonly, the reach downstream of a dam is characterized by sediment starvation, which can erode the bed and banks to regain some of its former sediment load. These erosive flows commonly induce incisions, undermine other infrastructure, and coarsen the bed^18^. In addition, sand mining has severe negative impacts on rivers, deltas, and coastal and marine ecosystems through, for example, the loss of land through rivers or coastal erosion, the lowering of water levels, and decreases in sediment supply, and it also affects socioeconomic development^19^. The scope of these adverse effects extends from the local area to more significant regions far from the sites where sand is taken. The natural sediment supplied from upstream regions cannot compensate for the extracted amount of sand, leading to a substantial annual deficit. The consequent sediment starvation within the water acts as a trigger for erosion processes. The downcutting of riverbanks can propagate upstream and downstream from extraction sites, affecting river bathymetry and ecosystems over a large area. As a result of the incision of river channels, bank erosion is strikingly exposed, resulting in coastline recession and increasing salinity intrusion.

**Figure 1:**
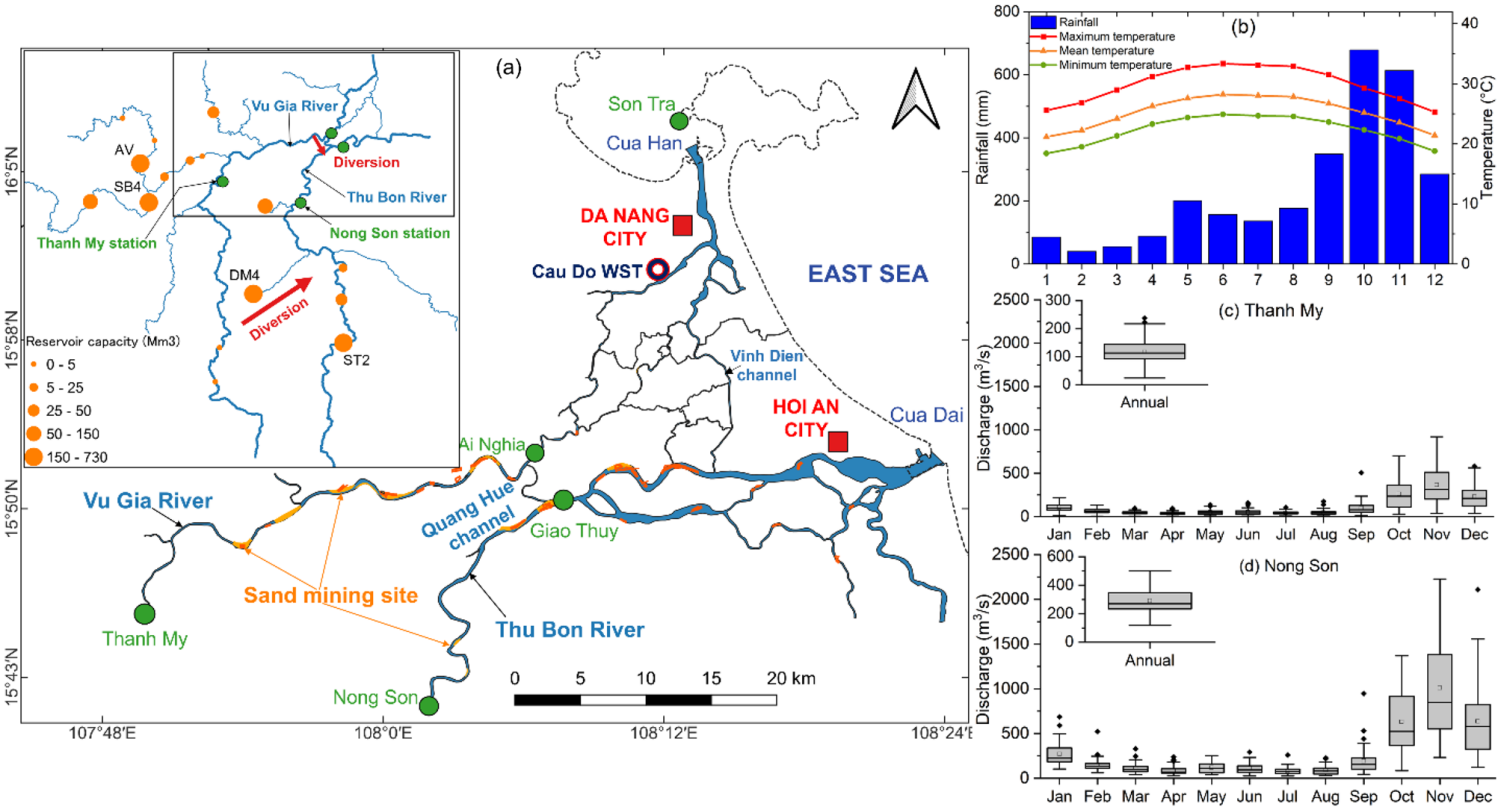
(**a**) Map of the VGTB River basin, (**b**) rainfall and temperature, (**c**) and (**d**) flow discharge at the Thanh My and Nong Son stations. Maps created in the QGIS version 3.28.4 (http://qgis.org/) and OriginPro 2023 (https://www.originlab.com/) softwares^20,21^.

The VGTB is a basin that has advantages in hydropower development and ranks 4th in terms of hydropower potential in Vietnam^22^ (Fig. 1a). The VGTB River basin is facing difficulties in maintaining its water supply and controlling saltwater intrusion, primarily due to the rapid development of artificial hydrostructures that alter the region’s hydrological dynamics^23,24^. Young dams, constructed since 2008 in the VGTB River basin downstream part region would take time for their impacts on the hydrological system to become visible. These dams modify the underlying hydrological regime, resulting in a concurrent shortage of sediment for the downstream region. The primary cause of changes in water resources for the Vu Gia and Thu Bon Rivers can be attributed to the transfer of water through the Dak Mi 4 hydropower plant and the Quang Hue channel^23^ (Fig. 1a). The Vu Gia River serves as the primary water supply source for Da Nang city and supports agricultural activities in the region. Water diversion has resulted in deficits in agricultural and drinking water supplies and increased salinity intrusion in Da Nang city^25,26^. Water shortages induced by saltwater intrusion, particularly during drought, significantly challenge the local population's domestic water supply and livelihood^27,28^. Thus, a controversial discussion between Da Nang city and Quang Nam Province has emerged, centering on the impact of hydropower development and water diversion and highlighting the complexities of water resource management in the region^23^. In contrast, the entire VGTB basin has only two stations measuring discharge, both of which are located upstream (Fig. 1a, c, d). The downstream tributaries, which are home to numerous hydropower plants, lack discharge observations, which poses a significant challenge in addressing this question. It is difficult to comprehensively investigate discharge, sediment, and reservoir impacts. Therefore, extending the estimated of discharge records for recent decades in this region is necessary to strengthen the scientific foundation for addressing this situation more effectively.

A full investigation of changes in the basin will help better manage planning. However, previous studies in the VGTB River basin have focused mainly on hydrology^26,29–31^, and geomorphological studies have been concentrated in the Cua Dai estuary^32,33^. These studies did not assess discharge, sediment, or river morphological changes from upstream to downstream^34,35^. Analysis and simulation use data from only two stations, so they cannot reveal dam impacts on streamflow and sediment^31,36^. Obviously, most past studies that analysed the sediment budget and morphological changes in the VGTB basin have not comprehensively analysed the sediment budget and morphological change due to a lack of data. In addition, the relationship between the headwater area and downstream area has not been determined, so the potential impacts from upstream to downstream areas may be quite large and serious. Therefore, this study aimed to collect data from many different approaches to comprehensively investigate the effects of anthropogenic activities on discharge, sediment budget, and morphological changes. The contents of this study are organized as follows: (1) investigate the discharge and sediment magnitude of the entire VGTB River basin using a semidistributed hydrological model (SWAT), (2) provide a complete understanding of the impacts of upstream anthropogenic developments on long-term discharge and sediment, (3) link reductions in sediment upstream and increase sand mining to spatiotemporal morphological changes, and (4) clarify the responses of water levels to streamflow alterations, sediment budgets, and morphological changes. The results of this study provide evidence and a reference for water resource and sediment management, hydropower development, and sand mining to quickly adapt to climate change and ensure sustainable development in the VGTB River basin.

### Study area

The VGTB basin has a tropical monsoon climate (approximately 10,350 km^2^). The rainfall is distributed unevenly across the basin, descending from the mountains to the plain coast. The average annual rainfall varies significantly from 2184 mm in the central and downstream regions to more than 4188 mm in the southern mountainous areas^24^ (Fig. 1b). Its upper part is short and steep, with a narrow riverbed, steep banks, and many cascades. The riverbed is relatively wide and shallow in the middle and downstream regions, respectively. The altitude, rugged terrain, and significant precipitation provided great potential for hydropower energy in the upper parts of the basin (Fig. 1a).

The basin is formed by two primary subbasins: the Vu Gia and Thu Bon subbasins. The river network is dense, and water is exchanged between Vu Gia and Thu Bon through the Quang Hue Channel and Dak Mi 4 hydropower plant (Fig. 1a). The VGTB basin interacts with the east sea directly through the Cua Han and Cua Dai mouths. Therefore, tidal features strongly affect the hydrological regime of estuaries.

According to the Department of Natural Resources and Environment and the Japan International Cooperation Agency (JICA), the total sand-mining volume during this period was 443 Mm^3^ from 1990 to 2007. The total annual sand-mining volume ranged from 0.3 to 3.2 Mm^3^/yr from 2008 to 2017. The amount of sand mined from the river from 2011 to 2017 was approximately 7.9 Mm^3^, with an annual average of 1.12 Mm^3^/yr.

**Figure 2:**
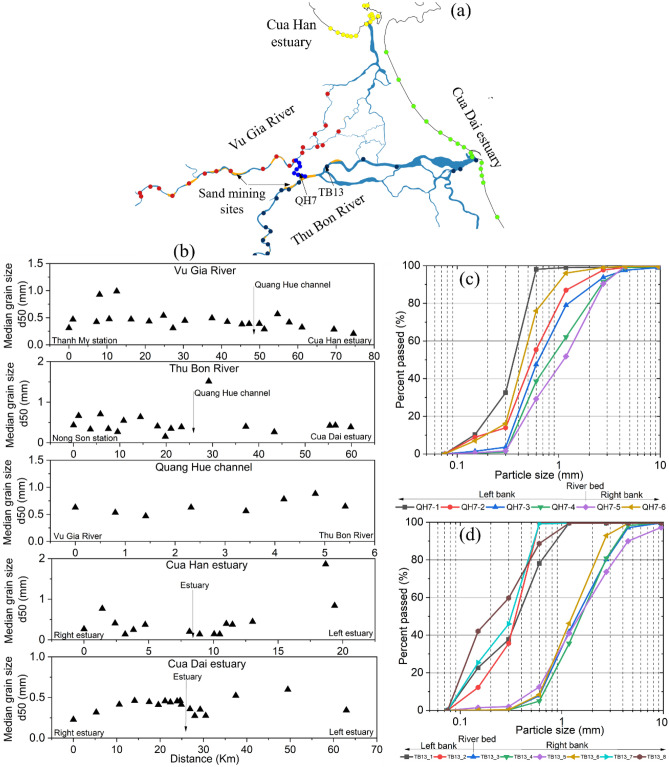
(**a**) General map of the Vu Gia Thu Bon River basin showing samples collected in the main river, estuaries, and along the beach (the red, navy blue, blue, yellow, and green circles represent sediment samples collected in the Vu Gia River, Thu Bon River, Quang Hue channel, Cua Han estuary, and Cua Dai estuary, respectively). (**b**) Mean grain size (d50) of the Vu Gia River, Thu Bon River, Quang Hue Channel, Cua Han in estuary, and Cua Dai estuary. (**c**–**d**) Grain size from the left bank to the riverbed and right bank at a cross-section in the Quang Hue channel (QH7) and the Thu Bon River (TB13). Maps created in the QGIS version 3.28.4 (http://qgis.org/) and OriginPro 2023 (https://www.originlab.com/) softwares^20,21^.

The experimental results of the grain size distribution showed that there was a significant difference in the grain size from upstream to downstream and for each cross-section in the VGTB River system (Fig. 2). In the upstream region of the Vu Gia River, the grain size was characterized by medium to coarse sand with d50 values of 0.31–0.99 mm, while the middle and downstream regions were mainly composed of medium sand with d50 values ranging between 0.21 mm and 0.57 mm (Fig. 2b). From upstream to downstream in the Thu Bon River, the grain size was characterized by fine to medium sand with a d50 of 0.15–0.71 mm. The grain size of the Quang Hue Channel changed little, and there was medium sand. In the cross-section, the grain size increased from the riverbed to the side bank (Fig. 2c, d). In the estuaries of the Vu Gia River (Cua Han) and Thu Bon River (Cua Dai), the grain size increases gradually from the outlet to the side-beach (Fig. 2b).

## Data and methodology

### Data collection

In this study, hydrometeorological, hydrological, hydraulic, topographic, bathymetric, landuse, soil type, infrastructure structure, and sand mining data were collected. The discharge and sediment data were collected from observations and from the SWAT model simulation results. Specifically, we collected discharge and sediment at Thanh My, Nong Son (upstream region), and data from the Ai Nghia and Giao Thuy locations in the middle of the basin were obtained from the SWAT model (Fig. 1a).

We selected two stations (Ai Nghia and Giao Thuy) in typical locations to analyse and clarify spatiotemporal water level changes. The Ai Nghia and Giao Thuy stations are located in the middle of the subbasins and downstream of the Quang Hue Channel. Therefore, we can assess the impact of upstream infrastructure and water diversion (Fig. 1a).

The field surveys were conducted in March 2021 along the two main rivers and the Quang Hue Channel, totaling approximately 240 km. The Thanh My station was measured from the Cua Han estuary (Vu Gia River), and the Nong Son station was measured from the Cua Dai estuary (Thu Bon River) (Fig. 1a). Bathymetric surveys were conducted using an acoustic Doppler current profiler (ADCP) and single-beam echosounder (Odom Hydrotrac II) accompanied by the Trimble R5 and R8 GPS system. Seventy-one cross-sections were intentionally measured at each site in 2010, 2015, 2018, and 2021.

We collected 98 samples of sediment from the riverbank along the VGTB River system and the Cua Han and Cua Dai estuaries to study the mechanism of the suspended sediment (Fig. 2a). The samples were collected on riverbeds, floodplains, roads, fields, near estuaries, and along the beach. Materials were collected from the cross-sections of the left bank, riverbed, and right bank of the riverbed. Approximately 1–2 kg of each sample was collected, stored in a plastic bag and labelled. Then the sediment samples were brought to the laboratory for futher experiments.

### Data processing and analysis

**Figure 3:**
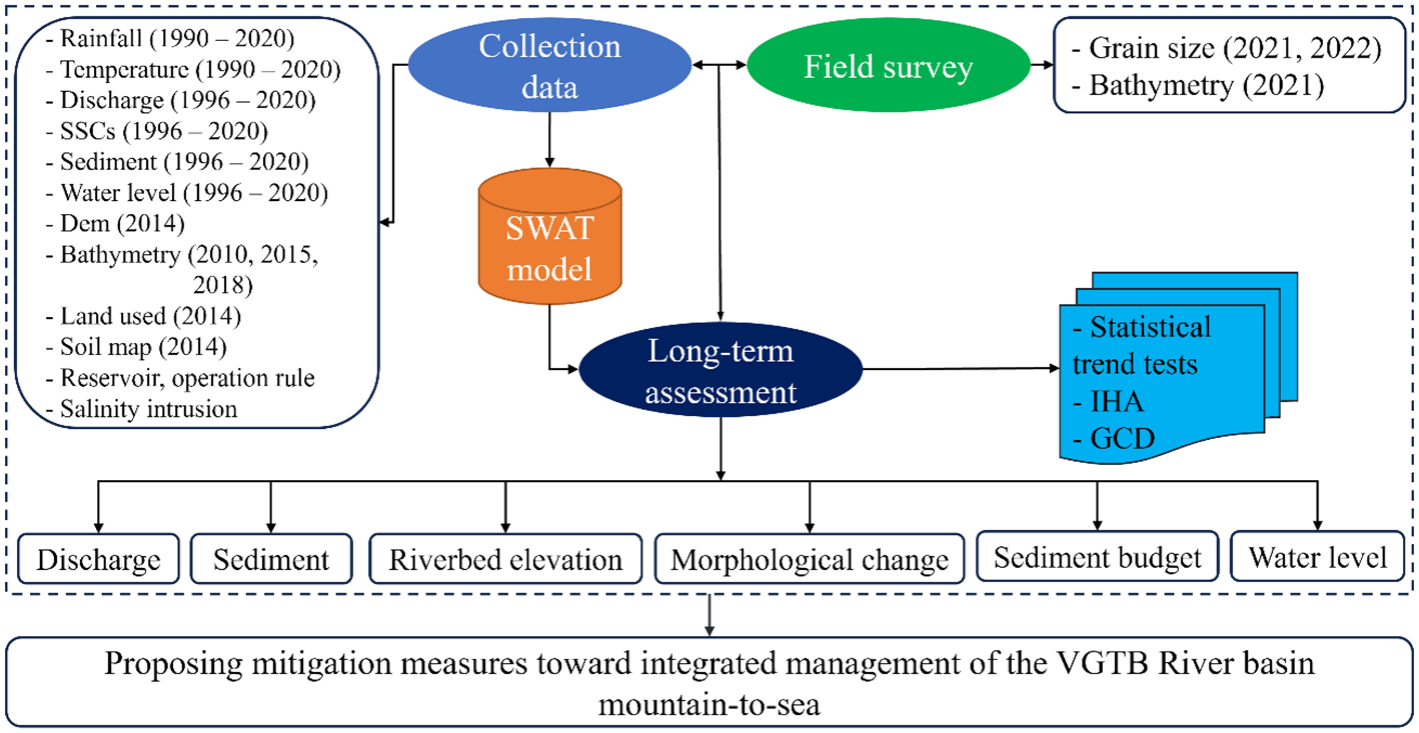
Schematic methodology framework of the research.

Changes in daily flow, sediment, and water level were investigated. First, we identified the trend and change point of discharge and sediment by Pettitt and used the nonparametric slope methods of Mann–Kendall and Sen (p = 0.05) to estimate the change rates^37–41^ (Fig. 3). Based on the change years, we divided the time series into two analysis predam (1996–2010) and postdam (2011–2020) periods. Hydrologic alteration (IHA) indicators were applied to quantify the difference between periods^42–44^. The IHA method used daily data and included thirty-two hydrologic indicators, which were categorized into groups according to magnitude, timing, duration, and frequency.

The Analyst module in ArcGIS®10.4.1, which includes deterministic and geostatistical methods with different parameters, was selected for analysing the 2021 bathymetric data. Universal kriging was identified as the best method, in addition to the exponential method with the kernel function. The selected method was applied to interpolate the 2010, 2015, and 2018 bathymetric data. To minimize distortions in the resulting values due to outliers and gaps in the bathymetry difference raster, the focal statistic tool at a 5-pixel window was chosen to fill and average the values via interpolation. The dataset was resampled via bilinear interpolation at a 10-m resolution before being clipped to match the study area.

The Geomorphic Change Detection (GCD) tool integrated with ArcGIS was used to quantify the geomorphic processes of erosion and deposition and estimate the sediment budget during the study period. GCD provides a suite of tools for measuring such uncertainties independently in each DEM and propagating to provide a DEM of differences. The program also provides ways to segregate the best spatial change estimates using different types of masks. The volumetric storage change was calculated from the surface elevation differences discerned from digital elevation models (DEMs)^45^.

### Semidistributed hydrological model SWAT

The entire VGTB basin has only two stations measuring discharge; both are located upstream. The downstream tributaries, which are home to numerous hydropower plants, lack discharge observations, which poses a significant challenge in addressing this question (Fig. 1a). Therefore, it is difficult to investigate discharge and reservoir impacts comprehensively. A semi-distributed hydrological model SWAT was selected to set up for the VGTB basin from 1990 to 2020^24,27^ (“Supplementary Information”). The calibration and validation periods in the warning model from 1990 to 1995 are 1996–2010 and 2011–2020, respectively.

The Soil and Water Assessment Tool (SWAT) is a time-based, semidistributed hydrological model developed and supported by the U.S. Department of Agriculture (USDA) and Agriculture Research Service (ARS)^46^. The SWAT model stores basin characteristics (DEM, land use, soils) and estimated runoff in minor spatial units known as hydrologic response units (HRUs). Runoff was calculated separately and stored in each HRU, after which subbasin and total basin runoff were calculated by summing them^46,47^. SWAT has been applied in many different watersheds to assess the impact of LULC, climate change, and other human activities on streamflow, sediment load, ecology, and the environment^10,48–50^. The SWAT model calculates daily surface runoff using the Soil Conservation Service (SCS) curve number (CN), a function of soil permeability, land use, and 5-day antecedent soil moisture content. For streamflow routing, the Muskingum method is used. Potential evapotranspiration (PET) was calculated by using the Penman–Monteith method. The hydrological cycle simulated by SWAT is based on the water balance equation^51^. The modified universal soil loss equation (MUSLE), a function of runoff factors, was used to predict sediment yield on a given day^52^. The MUSLE model was implemented in the SWAT model by assuming a simple hydrograph shape to estimate the daily runoff volume with a peak flow rate within the subwatershed area; this model was further used to predict the variation in runoff erosive energy. However, studies stress the need for further investigations of runoff curve numbers and the use of the Green-Ampt method for hydrologic data^53–55^. In addition, there is a need to continue testing and developing erosion estimation and sediment routing algorithms to suit different landscapes^56–58^.

The basin was divided into 153 subbasins and 2580 HRUs. The subbasins were categorized based on their slope classes, land classes, soil classes, hydrological stations, dam locations, water transfer and receiving sites, and uniform size distributions. The water was transferred from the Vu Gia subbasins to the Thu Bon subbasins via the Dak Mi 4 hydropower plant, and the Quang Hue channel was also established in the model^24^.

The calibration process for daily streamflow simulation was conducted using the Sequential Uncertainty Fitting algorithm version 2 (SUFI-2)^59,60^ within the SWAT-CUP program (version 5.2.1)^61^. For this study, nineteen primary parameters known for their high sensitivity within the SWAT model were selected^62^. The calibration process was conducted to select the most accurate fitted values for each scenario, the calibration process was conducted with a total of 500 simulations, and the validation process also involved 500 simulations. The Nash–Sutcliffe efficiency (NSE) was employed as the objective function to evaluate the model performance, ensuring a robust and accurate calibration and validation process for the SWAT model.

**Figure 4:**
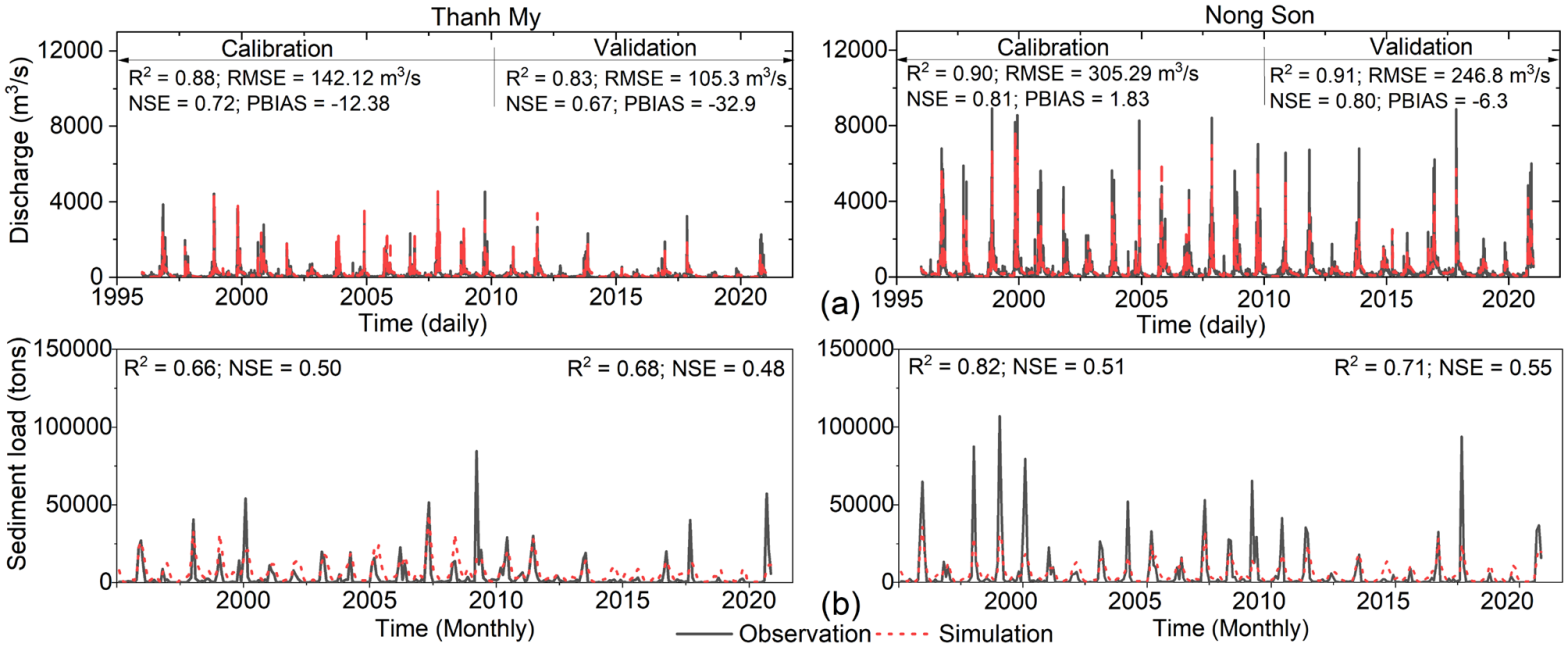
Calibrated and validated hydrographs of the discharge and sediment at (**a**) the Thanh My station and (**b**) the Nong Son station; calibrated (1996–2010) and validated (2011–2020).

The simulated flow of the model shows good agreement with the observed data in both the calibration and validation periods. The four performance evaluation criteria show the quality of the simulation model for the VGTB. The R^2^, NSE, RMSE, and PBIAS coefficients at the Thanh My and Nong Son stations during the validation period were 0.83, 0.67, 105.3 m^3^/s, and − 32.9; 0.91, 0.80, 246.8 m^3^/s, and − 6.3, respectively (Fig. 4a). For sediment simulation, the efficiency is lower than that for streamflow. There was also good agreement between the simulation and observation results during the calibration and validation periods at both stations (Fig. 4b). Therefore, the model is suitable for investigating the variation in discharge and sediment under the effect of anthropogenic activities.

## Results

### River discharge characterization

**Figure 5:**
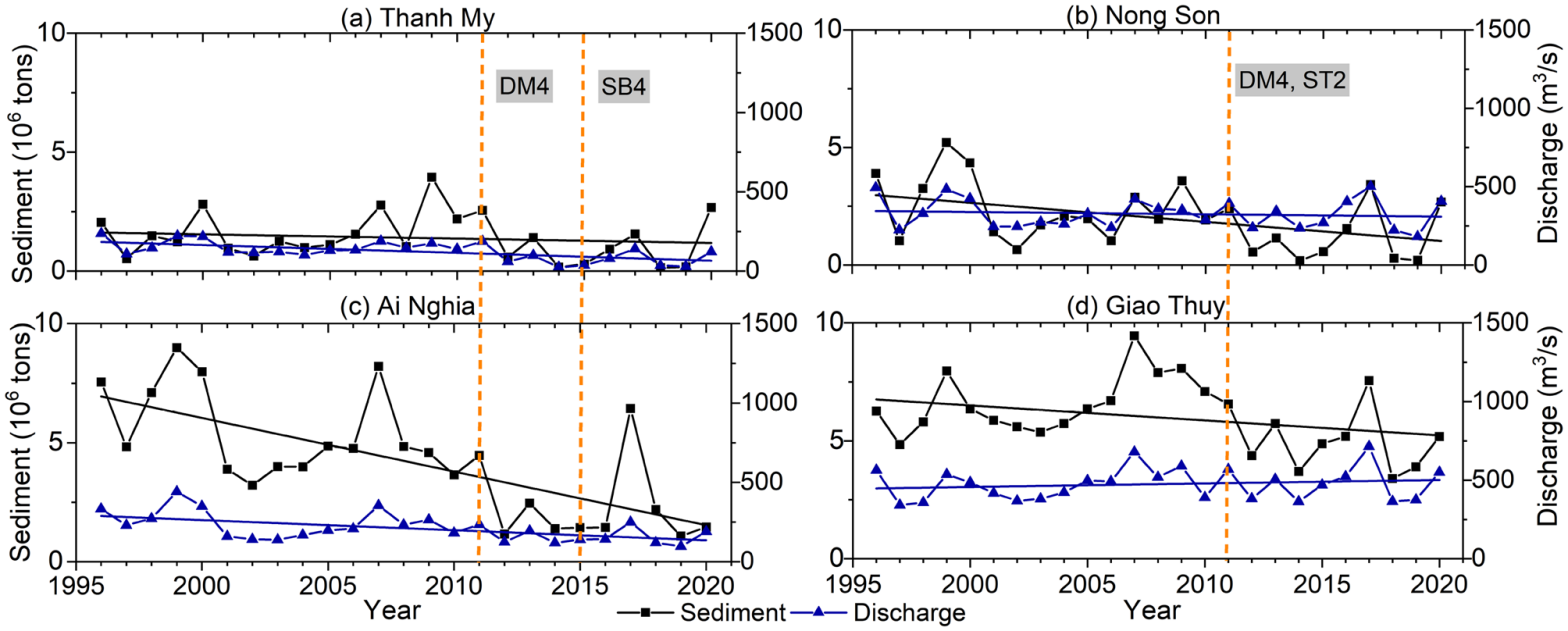
The long-term annual discharge and sediment load at the Thanh My, Nong Son, Ai Nghia, and Giao Thuy stations from 1996 to 2020 (DM4: Dak Mi 4 reservoir; ST2: Song Tranh 2 reservoir; SB4: Song Bung 4 reservoir).

**Figure 6:**
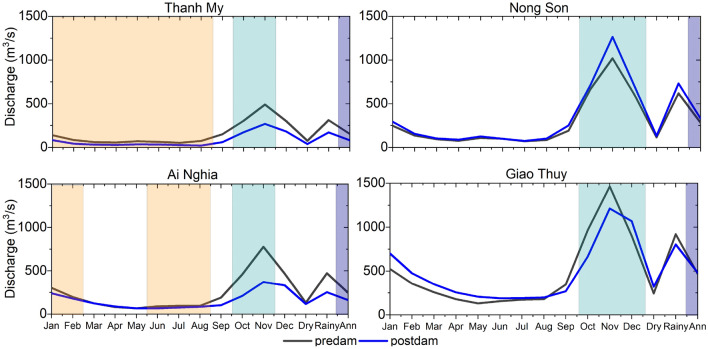
Monthly discharge in the predam (1996–2010) and postdam (2011–2020) periods at the Thanh My, Ai Nghia, Nong Son, and Giao Thuy stations. The highlights show the months with significant changes in the two periods. Yellow, green, and violet indicate significant changes in the dry season, flood season, and annually, respectively.

Figure 5 shows that the VGTB River discharge changes significantly during the postdam period compared to the predam period. The general trend is a decreased annual discharge in the Vu Gia River and increased discharge in the Thu Bon River. The annual discharge at the Thanh My, Ai Nghia, Nong Son, and Giao Thuy stations in the predam and postdam periods is 154 m^3^/s, 245 m^3^/s, 283 m^3^/s, and 469.5 m^3^/s, respectively, and at 82 m^3^/s, 160.9 m^3^/s, 330.8 m^3^/s, and 481.7 m^3^/s, respectively (Table 1, Fig. 6).

**Table 1:** The results of the IHA analysis determined discharge alterations at the Thanh My and Nong Son stations.

Indicator	Units	Thanh My station			Nong Son station		
		Predam	Postdam	Deviation magnitude (%)	Predam	Postdam	Deviation magnitude (%)
January	m^3^/s	120	49	− 71(− 59)	227	297	70(31)
February	m^3^/s	81	23	− 58(− 72)	148	167	19(13)
March	m^3^/s	57	13	− 44(− 77)	99	148	48(49)
April	m^3^/s	47	16	− 31(− 66)	72	112	40(55)
May	m^3^/s	57	19	− 38(− 67)	93	154	60(64)
June	m^3^/s	48	11	− 37(− 77)	87	135	48(56)
July	m^3^/s	44	10	− 34(− 77)	64	104	40(63)
August	m^3^/s	59	11	− 48(− 81)	69	101	32(47)
September	m^3^/s	89	24	− 65(− 73)	113	132	19(17)
October	m^3^/s	193	50	− 143(− 74)	381	294	− 87(− 23)
November	m^3^/s	262	90	− 172(− 66)	696	573	− 124(− 18)
December	m^3^/s	209	83	− 126(− 60)	483	587	104(22)

We found that the annual discharges at Nong Son and Giao Thuy increased, while the sediment decreased sharply, especially at Nong Son, according to the Mann–Kendall test (Fig. 5). On the other hand, discharge and sediment decreased at Thanh My and Ai Nghia, especially at Ai Nghia. The change point occurred in 2011, as detected by the Pettitt test. The sediment concentration decreased significantly at the same time that the Dak Mi 4 Song Tranh 2 reservoirs were completed in the Vu Gia and Thu Bon Rivers (Figs. 1a, 5).

Due to anthropogenic intervention in the postdam period, the annual discharge of Thanh My decreased by 68%, and that of Nong Son increased by 11% (Table 1). During the postdam period, the annual impact variation in the dry season (January–August) increased slightly in Nong Son but decreased considerably in Thanh My (Table 1). At Thanh My, the percentage of rates decreased by 59–81%, and at Nong Son, the percentage increased by 13–64%. The diversion of Dak Mi 4 alternately decreased the mean daily streamflow in individual years in Vu Gia. As a result, the dam regulation increases the expected flow variability and the frequency of low flow. The general trend decreased at four stations in the flood season (September–December) (Table 1, Fig. 6).

### Long-term spatiotemporal alterations in sediment load

**Figure 7:**
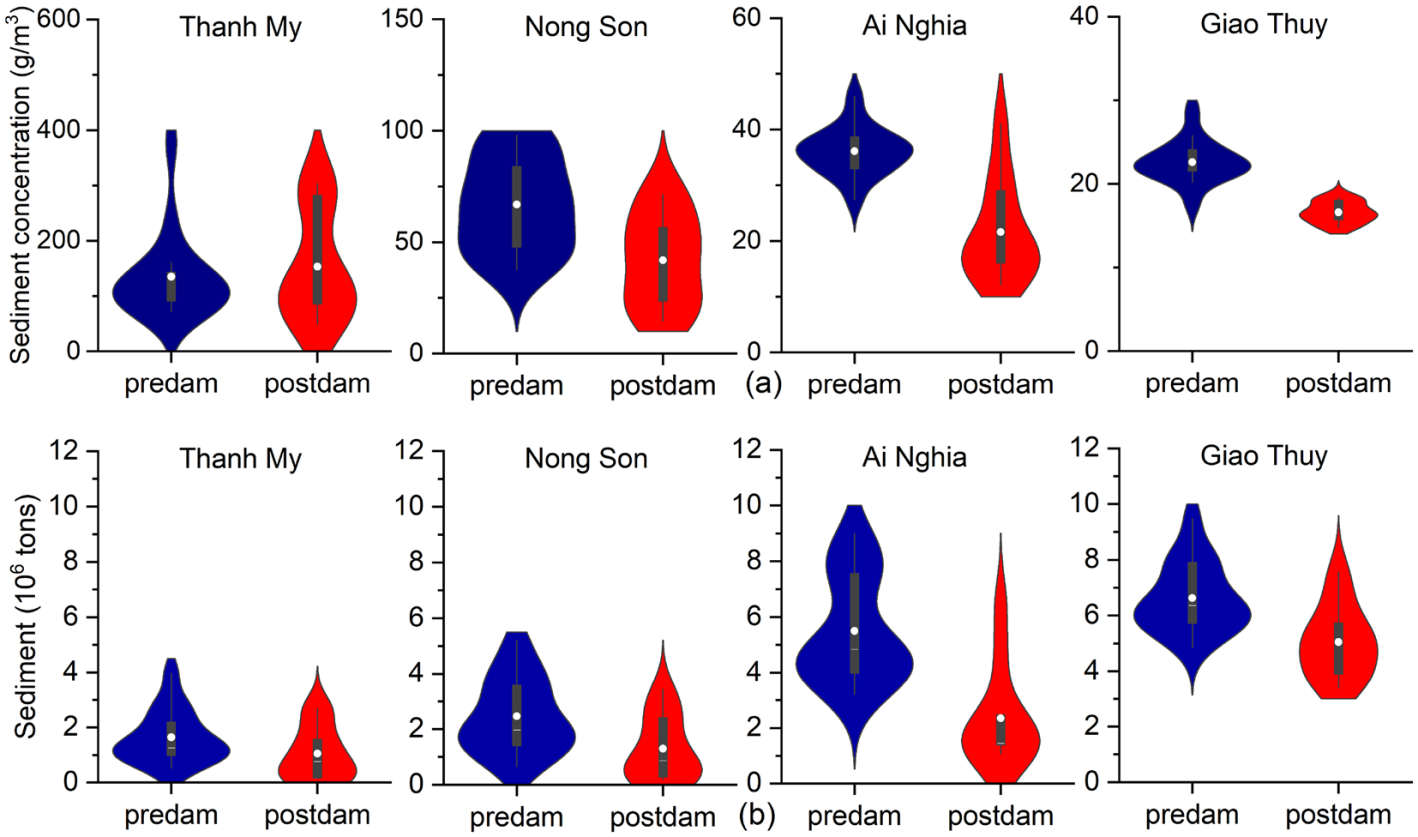
The violins represent kernel density plots of the daily suspended sediment concentration (**a**) and annual sediment concentration (**b**) at the Thanh My, Nong Son, Ai Nghia, and Giao Thuy stations in the predam and postdam periods. The black lines represent box-whisker plots, the white points represent the means, and the white lines represent the medians.

Box plots of the daily suspended sediment concentration (SSC) showed that the mean daily SSC decreased significantly from the predam period to the postdam period at Nong Son, Ai Nghia, and Giao Thuy (Fig. 7a). The SSC decreased by 12.2 g/m^3^, 15.3 g/m^3^, and 5 g/m^3^, respectively. However, Thanh My station increased from 135 to 153 g/m^3^. The data spread is more significant in the predam period than in the postdam period. In addition, the higher probabilities of the four stations are concentrated in the median value during the postdam period.

We found that the annual sediment amount decreased at four stations during the postdam period compared with that during the predam period (Fig. 7b). When Ai Nghia decreased the most, the mean annual sediment decreased by 57.3%, from 5.5 to 2.35 million tons. Although sediment was received from the Vu Gia River at two locations, the mean annual sediment at Giao Thuy still decreased by 1.58 million tons (23.8%), from 6.63 to 5.05 million tons. The mean annual sediment also decreased at Thanh My and Nong Son by 0.34 and 1.41 million tons, respectively. This result was in agreement with the values reported by JICA in 2018. After dam construction, the sediment volume was reduced by 1.4 million tons, compared to that before dam construction^64^. In addition, the sediment decreased at the Thanh My and Ai Nghia stations on the Vu Gia River because of the flow discharge and sediment diversion from the Dak Mi 4 hydropower plant and Quang Hue channel^23,24,27,28^ (Fig. 1a).

### Effects of reducing sediment and sand mining on bathymetry

**Figure 8:**
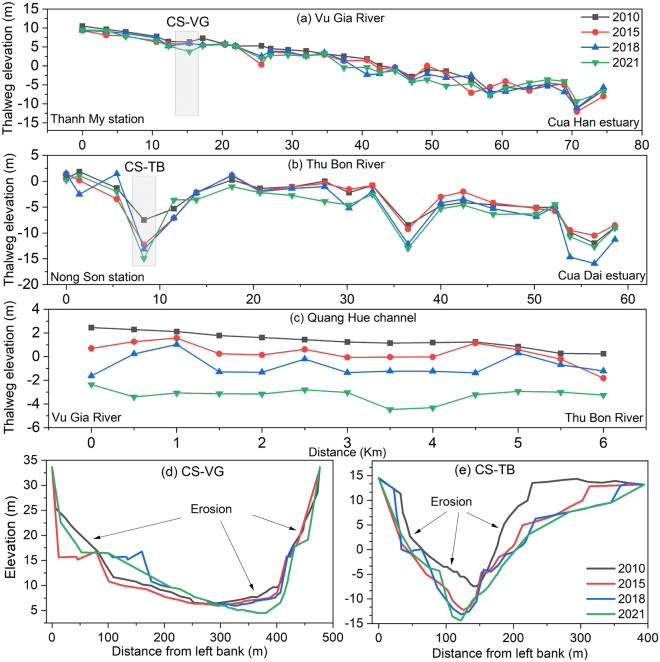
(**a**) Thalweg elevation of the Vu Gia River from Thanh My station to the Cua Han estuary, (**b**) Thalweg elevation of the Thu Bon River from the Nong Son station to the Cua Dai estuary, (**c**) Thalweg elevation of the Quang Hue channel, (**d**) Elevation of cross-section CS-VG on the Vu Gia River, (**e**) Elevation of cross-section CS-TB on the Thu Bon River.

Figure 8 plots the thalweg elevation in 2010, 2015, 2018, and 2021 along the Vu Gia, Thu Bon Rivers, and Quang Hue channels. We found that the period with widespread human activity was 2018–2021, which was different from the 2010–2015, and 2015–2018 periods. The effects of dams on morphological changes extend and shift progressively downstream. The riverbed elevation changes from 68 and 74 km from downstream dams on the Vu Gia and Thu Bon Rivers, respectively (Fig. 8a, b). The effective distances to the delta were 98 km and 94 km, respectively.

Sand mining sites are concentrated in the middle of the basin and close to the delta. Sand mining removes large quantities of riverbed sediments and creates numerous pits and pools. By analysing cross-sectional and longitudinal profiles obtained during our 2021 survey distinct sand mining pockmarks, were found of reach depths of up to 7.1 m (~ 36 km Vu Gia River), 4.2 m (~ 27 km Thu Bon River), and 5.5 m (~ 5.0 km Quang Hue channel) compared with those in 2010 (Fig. 8a–c). Reducing sediment from upstream and large sand mining downstream leads to a decrease in riverbed bathymetry in the downstream Vu Gia and Thu Bon Rivers. Generally, the riverbed elevation of the VGTB River system decreases over time. The average thalweg elevation decreased from 2010 to 2021 along the Vu Gia, Thu Bon, and Quang Hue channels by 0.98 m, 1.45 m, and 4.62 m, respectively (Fig. 8a–c). This also occurs in cross-sections, for example, CS-VG on the Vu Gia River and CS-TB on the Thu Bon River (Fig. 8d, e).

A comparison of the cross-sectional and longitudinal profiles from 2010 and 2021 reveals distinct signs of sand mining (Fig. 8e). There were notable irregularities in the riverbed in 2021. The observed disparities in these cross-sectional and longitudinal profiles provide substantial evidence of ongoing and intensifying sand mining operations in the basin, especially during the period from 2018–2021. This has had a significant impact on the riverbed elevation.

### Effects of hydropower dams and sand mining on morphological changes and sediment budgets

**Figure 9:**
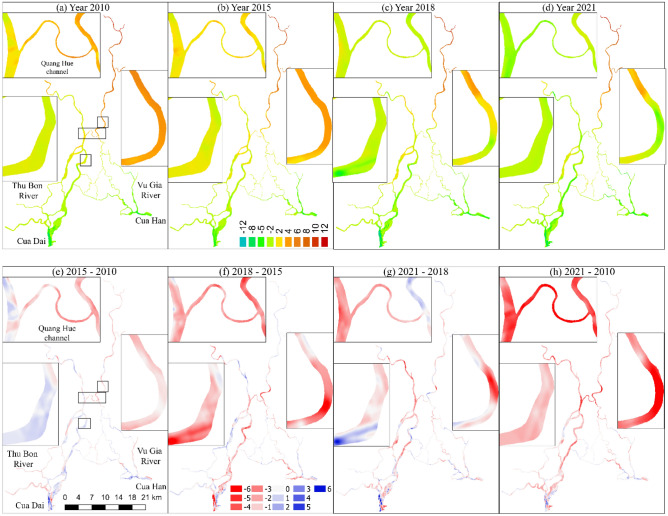
(**a**–**d**) The riverbed elevation in 2010, 2015, 2018, and 2021. (**e**–**h**) The riverbed elevation differences in the 2015–2010, 2018–2015, 2021–2018, and 2021–2010 periods. The units of riverbed elevation and riverbed elevation differences are meter. Maps created in the QGIS version 3.28.4 (http://qgis.org/) softwares^21^.

**Figure 10:**
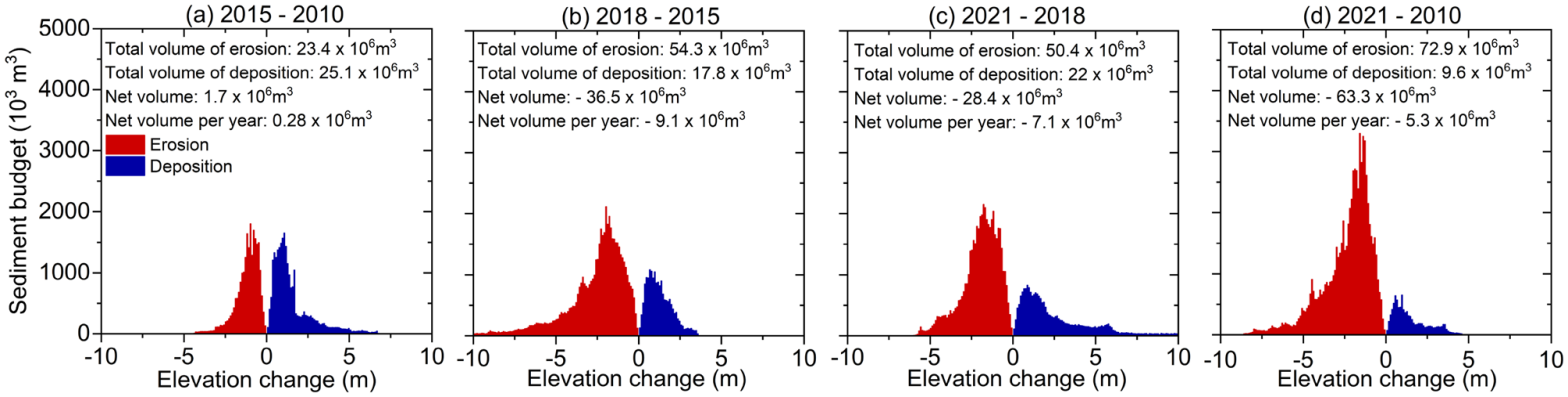
Diagram of the sediment budget by elevation change in the (**a**) 2015–2010, (**b**) 2018–2015, (**c**) 2021–2018, and (**d**) 2021–2010 periods.

The sediment budget exhibited a balance between erosion and deposition during the period 2015–2010 (Figs. 9e, 10a). However, the riverbed elevation and sediment budget significantly changed between 2018 and 2015 and between 2021 and 2018. The riverbed is mostly eroded. The net annual volume is 9.1 Mm^3^ and 7.1 Mm^3^, respectively (Figs. 9f, 9g, 10b, c).

A Comparison of riverbed elevation over 12 years between 2010 and 2021 revealed that the VGTB River system exhibited severe net riverbed incision. Figures 9a, d, h and 10d indicate that the recent sediment budget of the VGTB has a net deficit. We estimated the total incision volume during the period from 2010–2021 in our river sector (63.3 Mm^3^), in which the Vu Gia and Thu Bon Rivers accounted for 12.2 Mm^3^ and 33.0 Mm^3^, respectively.

For Vu Gia, riverbed incisions occurred in the middle and head downstream areas, and the erosion points were located on straight river sections. For Thu Bon, the erosion points developed strongly downstream, and erosion occurred in the bend areas (Figs. 8, 9). Significant incisions were recorded along the Vu Gia, Thu Bon River, and Quang Hue channels, which varied widely between periods. There seems to be a clear indication of accelerated incisions in recent years. The results presented in this study provide compelling evidence that sand mining is a major contributing factor instead of an upstream dam (Figs. 7, 8, 9, 10). This process drives riverbed changes and sediment budgets in the VGTB River. This finding is also consistent with the research of JICA (2018). Through detailed comparisons of riverbed elevation in three periods, incisions by sand mining were clearly distinguished in the VGTB River.

### Long-term spatiotemporal alterations in water level

During the dry and flood seasons, the water level decreased at Ai Nghia and Giao Thuy (Table 2). The water level decreased by 21.1% at Ai Nghia and 44.3% at Giao Thuy. The annual water level decreased by 0.557 m (15.1%) and 0.832 m (38.2%). The annual daily minimum decreased from the predam to the postdam at these stations. Especially at Giao Thuy, the annual daily minimum water level sharply decreased by 93.3%, from 1.051 to 0.07 m.

**Table 2:** Changes in the water level between the predam and postdam periods at the Ai Nghia and Giao Thuy stations.

Indicators	Ai Nghia station			Giao Thuy station		
	Predam (m)	Postdam (m)	Deviation magnitude (%)	Predam (m)	Postdam (m)	Deviation magnitude (%)
January	3.712	3.079	− 0.633(− 17)	2.418	1.511	− 0.907(− 37.5)
February	3.232	2.715	− 0.517(− 16)	1.858	1.101	− 0.757(− 40.8)
March	3.001	2.677	− 0.324(− 10.8)	1.534	0.966	− 0.568(− 37.1)
April	2.958	2.646	− 0.312(− 10.5)	1.421	0.857	− 0.564(− 39.7)
May	3.236	2.867	− 0.369(− 11.4)	1.633	0.964	− 0.669(− 41)
June	3.111	2.860	− 0.251(− 8.1)	1.519	0.931	− 0.588(− 38.7)
July	3.012	2.785	− 0.228(− 7.6)	1.344	0.824	− 0.52(− 38.7)
August	3.329	2.742	− 0.587(− 17.6)	1.497	0.834	− 0.663(− 44.3)
September	3.971	3.135	− 0.836(− 21.1)	2.073	1.154	− 0.919(− 44.3)
October	4.799	3.878	− 0.92(− 19.2)	3.330	1.958	− 1.372(− 41.2)
November	5.191	4.351	− 0.839(− 16.2)	4.107	2.752	− 1.355(− 33)
December	4.586	3.728	− 0.858(− 18.7)	3.402	2.308	− 1.094(− 32.2)
Annual	3.680	3.123	− 0.557(− 15.1)	2.179	1.347	− 0.832(− 38.2)
1-day minimum	2.564	1.550	− 1.014(− 39.5)	1.051	0.070	− 0.981(− 93.3)
1-day maximum	8.862	9.670	0.808(9.1)	8.153	9.030	0.877(10.8)

## Discussion

### The role of sand mining and cascade dams in riverbed incisions

The annual discharges at Nong Son and Giao Thuy increased, while the sediment decreased sharply, especially at Nong Son (Figs. 5, 6, 7; Table 1). We also note that these two locations in the Thu Bon River receive streamflow and sediment from the Vu Gia River. On the other hand, discharge and sediment decreased at Thanh My and Ai Nghia (Figs. 5, 6, 7; Table 1). The sediment content sharply decreased at Ai Nghia because the Quang Hue Channel transferred most of the sediment to the Thu Bon River. In addition, sediment significantly decreased when reservoirs were built in the upstream basin (for example, A Vuong, Song Bung 4, Dak Mi 4, and Song Tranh 2) (Figs. 5, 7).

Da Nang city and Quang Nam Province are experiencing rapid economic development^65^. The amount of sand material available for infrastructure is enormous and is mainly mined from the VGTB River system. Discharge and sediment reduction combined with sand mining activities, diversion drive riverbed incisions, morphological changes, and water level changes downstream (Table 2). The riverbed incision in the Vu Gia and Thu Bon Rivers started in 2011 when reservoirs began operating and sand mining increased (Figs. 8, 9). The thalweg elevation decreased at Ai Nghia and Giao Thuy between 2010 and 2021 by 1.8 m and 3.9 m, respectively (Fig. 8a, b). Morphological changes can damage structures along riverbanks, affect essential water availability for public water supplies and irrigation, and increase flood risk. Field surveys show that sixty-eight hot spots are distributed from upstream to downstream. Erosion sites were mainly found in the sand mining area and at the intersection of river tributaries according to the 2021 field survey.

### Linking change in bathymetry to the water level

**Figure 11:**
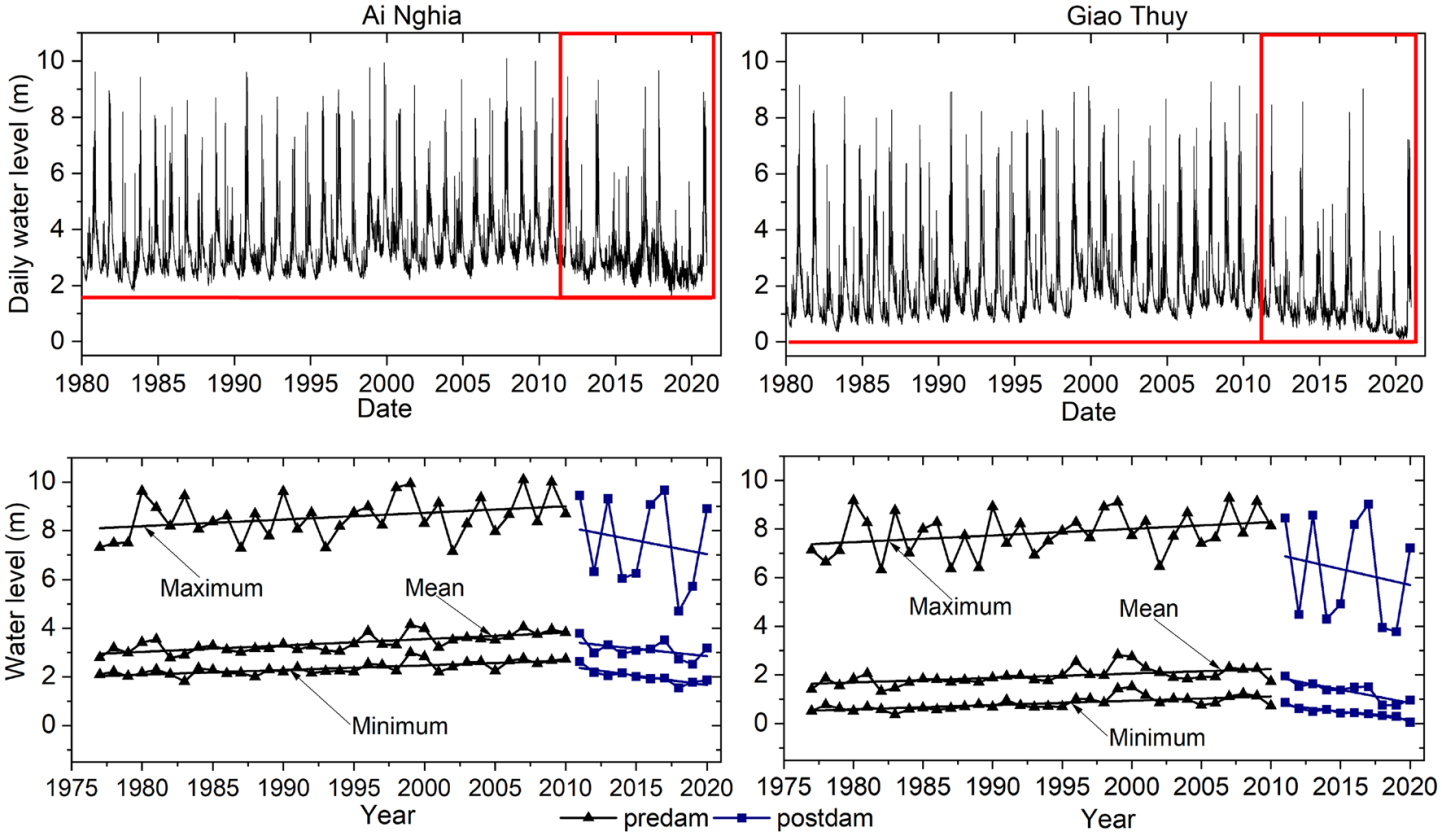
Daily water level and the maximum, mean, and minimum water level trends at the Ai Nghia and Giao Thuy stations.

The monthly and annual minimum water levels at Giao Thuy decreased, although the corresponding discharges increased (Tables 1, 2, Figs. 5, 6). Dam operations cannot explain these decreases in the water level due to the increased dry season water level through increased discharge. Irrigation expansion is likely not the cause because it did not reduce the dry season discharge of the Thu Bon subbasin. Moreover, the Thu Bon River also receives water from the Vu Gia River via Dak Mi 4 and Quang Hue. Giao Thuy is located in the middle of the basin and is not affected by tides (Fig. 1a). Therefore, we argue that these decreases in the water level are mainly driven by riverbed incision caused by the decreased sediment load and accelerated sand mining (Figs. 8, 9, 10). Sand mining occurs in large quantities in the middle and downstream areas (from the Giao Thuy station to the outlet). Thalweg elevation in this river section has greatly decreased (Figs. 8b, 9). This approach is suitable for decreasing in the annual mean water levels at Giao Thuy starting in 2011 (Table 2, Fig. 11).

The annual daily minimum and monthly water levels decreased at the Ai Nghia station (Table 2, Fig. 11). Riverbed incision also occurs downstream of Vu Gia due to the decreased sediment load and accelerated sand mining (Figs. 8a, 9). In addition, part of the streamflow and sediment diverted to the Thu Bon River also affects the bathymetry and water level downstream.

### Linking changes in discharge and water level reduction on saline intrusion, water supply, and agricultural production

Changes in discharge patterns resulting from anthropogenic activities such as hydropower development and water diversion can lead to severe consequences. In addition, river deepening can also trigger and increase salinity intrusions and subsequently affect the water supply and agricultural production^8,15^. Saltwater intrusion-induced water shortages during drought are the main constraints hindering the domestic water supply and agricultural production. Additionally, the outlet of the VGTB basin experiences a semidiurnal tidal regime, with the water level rising and falling twice daily. Therefore, downstream flow is frequently affected by saline intrusion during the dry season. In recent years, saltwater intrusion has become a significant problem in Da Nang city, particularly at the outlet of the Vu Gia basin. Monitoring data from the Da Nang Water Supply Joint Stock Company (DAWACO) indicate that there has been an increase in the maximum salt intrusion and the number of days with salinities above the threshold since the operation of reservoirs in 2012. In addition, saltwater intrusion is expected to be exacerbated by climate change and rising sea levels. The data observed from 1983 to 2020 at the Son Tra oceanographic station (Fig. 1a), which is located at the outlet of the Vu Gia Basin show that sea level has increased 3.3 mm/year. Considering the impacts of climate change, sea level rise, riverbed incision, and the operation of upstream dams, saltwater intrusion in the downstream VGTB basin is predicted to worsen both in frequency and magnitude during the dry season^26,66^.

Economic development and rising populations increase energy demand and natural resources, including water resources. With the rapid increase in urbanization in the study area, the demand for water supplies for living and production will continue to increase. Water resources have become more vulnerable due to hydraulic infrastructure, water transfer, and climate change. Typically, salinization at the Cau Do water plant has caused stress in the domestic water supply to Da Nang city (Fig. 1a). Salinity in the Vinh Dien Channel affects pumping stations serving agricultural irrigation in part of Quang Nam Province. A similar situation has also been found in the Mekong and Red River deltas^67–70^.

Agriculture is an important economic sector of the basin, with 70% of irrigated agriculture being paddy rice^26^. Therefore, agricultural production in the VGTB basin may become increasingly vulnerable due to interrupted irrigation. Analysis of land use data from 2010 to 2020 showed that rice areas gradually decreased by 11.8%. These findings provide a scientific basis for future management plans of stakeholders and decision-makers regarding water resource management in the VGTB basin, especially for sustainable agricultural development.

### Proposing mitigation measures for the integrated management of the VGTB River basin mountain-to-sea

There is an inseparable biological link between the general ecosystem and the water sources of the river basin, the coastal zone, and the sea^71–74^. The interaction scale, however, depends on the scale and morphologic characteristics of the river basin.

**Figure 12:**
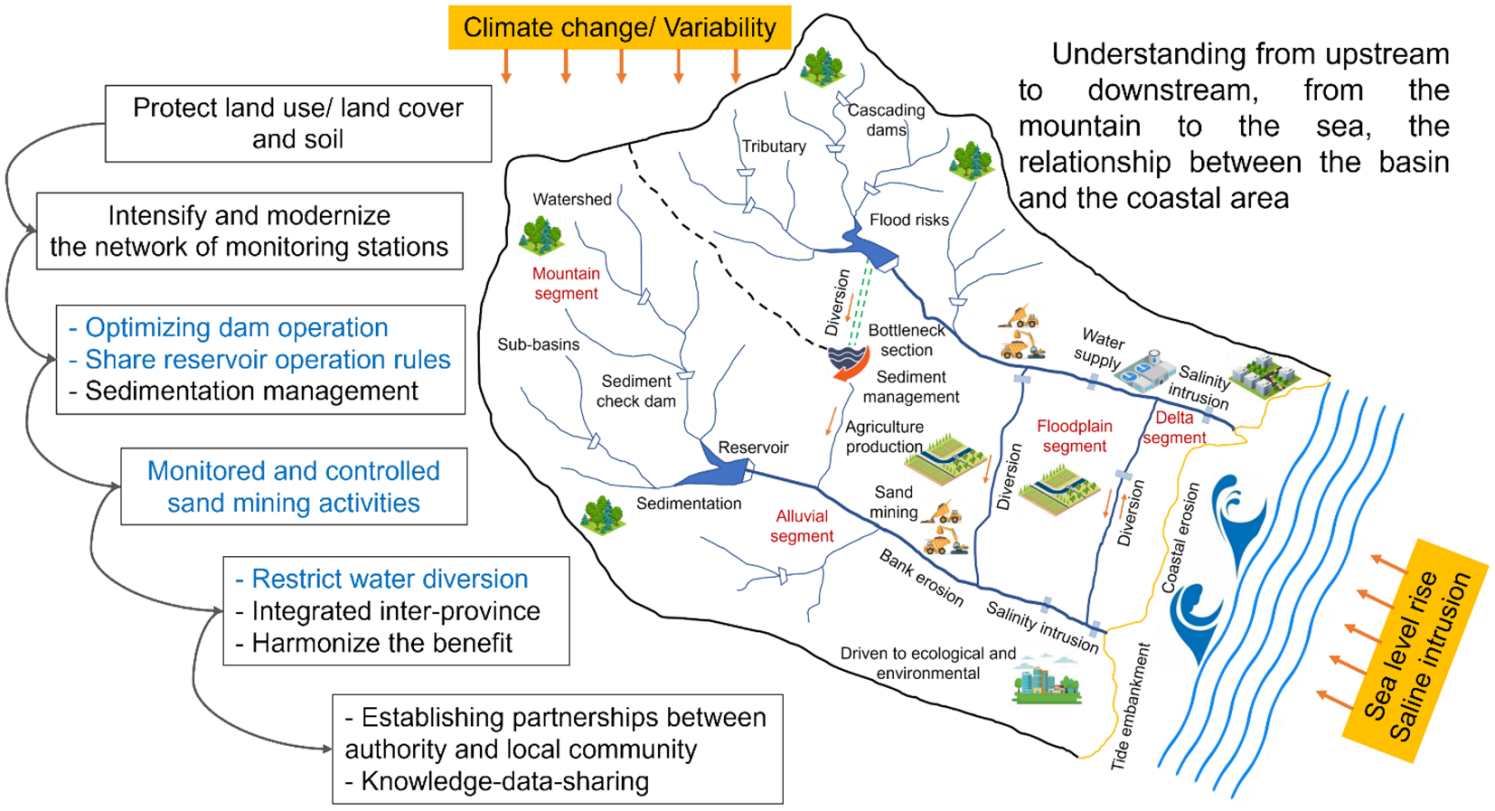
Mitigation measures should be proposed for the integrated management of the VGTB River basin.

The results showed that anthropogenic and climate variability severely impact the VGTB River basin. Therefore, an approach for integrated water resource management in river basins and coastal zones in needed to ensure sustainable development in the VGTB River basin. This will then enable the integration of policies, development plans, and adaptive solutions for river basins and coastal zones (Fig. 12). Strengthened coordination is needed across sectors, incentives, and institutionalizations of the participation of related stakeholders as well as the community in the field of river basins and coastal zones. Selecting and developing regional (interregional) linkages are the basis for tackling and mitigating the impacts of river basins on coastal areas, as well as the impacts of coastal areas on the sea^75^.

## Conclusion and recommendations

We applied different statistical methods and numerical models (SWAT) to assess long-term spatiotemporal changes in sediment load from 1996 to 2020. River bathymetric data (from 2010, 2015, 2018, and 2021) were also investigated further to clarify the impact of upstream dams and sand mining. We found that dam development, sand mining, and land use changes are the main drivers for flow discharge and sediment alterations. Morphological changes have increased the water transfer rate from the Vu Gia River to the Thu Bon River through the Quang Hue Channel. This has not only affected the water supply but also increased the risk of saltwater intrusion. As a result, water shortages induced by saltwater intrusion during drought periods have emerged as a significant constraint in hindering the domestic water supply and agricultural production. These results can thus provide a scientific basis for policymakers and decision-makers to design and implement effective and sustainable water management plans for the VGTB basin. Our findings are summarized as follows:

1. The annual discharge at Nong Son and Giao Thuy increased, while the sediment decreased sharply. On the other hand, discharge and sediment decreased at Thanh My and Ai Nghia. Similarly, compared with those in the predam period, the annual sediment in the Vu Gia and Thu Bon Rivers decreased by 57.3% and 23.8%, respectively, in the postdam period. Reducing sediment from upstream areas and large sand mines downstream led a decrease in riverbed bathymetry in the VGTB River system from 2010 to 2021. The thalweg elevation decreased at Ai Nghia and Giao Thuy between 2010 and 2021 by 1.8 m and 3.9 m, respectively.
2. We also found that riverbed incisions occur downstream of the Vu Gia and Thu Bon Rivers by reducing the sediment load and accelerating sand mining. The total incision volume during the period of 2010–2021 in our concerned river sector was 63.3 Mm^3^, in which the Vu Gia and Thu Bon Rivers accounted for 12.2 Mm^3^ and 33.0 Mm^3^, respectively.
3. Discharge and sediment reduction drivers of morphological changes and water level changes downstream. The water level decreased by 21.1% at Ai Nghia and 44.3% at Giao Thuy. The annual water level decreased by 0.557 m (15.1%) and 0.832 m (38.2%).

A complex river network connects the VGTB River basin. Therefore, to investigate the basin in detail, especially under the impact of climate change and anthropogenic activities, it is necessary to collect data and establish a full hydro-sediment-morphodynamics model. From there, we can fully understand the effects of cascade dams, sand mining, and diversion on flow discharge, sediment budget, and morphological change.

### Supplementary Information

Supplementary Information.

## Data Availability

The DEM, land use, and soil maps were obtained from the website of the “Land Use and Climate Change Interaction in Central Vietnam”—(LUCCi) project (www.lucci-vietnam.info). The datasets used and analysed during the current study are available from the corresponding author upon reasonable request.
