# Supplementary material for: Quantifying the consequences of unsustainable sand mining and cascade dams on aspects in a tropical river basin
Source: Sci Rep. 2024 Jan 12;14:1178. doi: 10.1038/s41598-024-51405-z (PMC10786850; doi:10.1038/s41598-024-51405-z)
Supplement: Supplementary file 1 — Supplementary Information. [file 41598_2024_51405_MOESM1_ESM.docx]

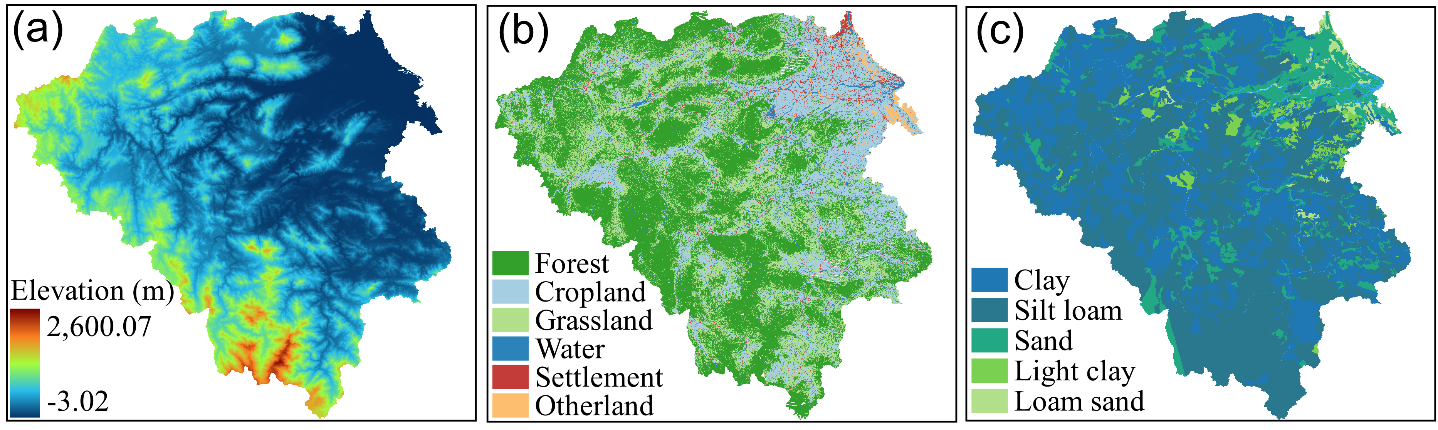


**Figure S1.** (a) The digital elevation model (DEM), (b) Land use map, and (c) soil map. The DEM, land use, and soil maps were obtained from the website of the “Land Use and Climate Change Interaction in Central Vietnam” - (LUCCi) project (www.lucci-vietnam.info). Maps created in the QGIS version 3.28.4 (<http://qgis.org/>) softwares^21^.


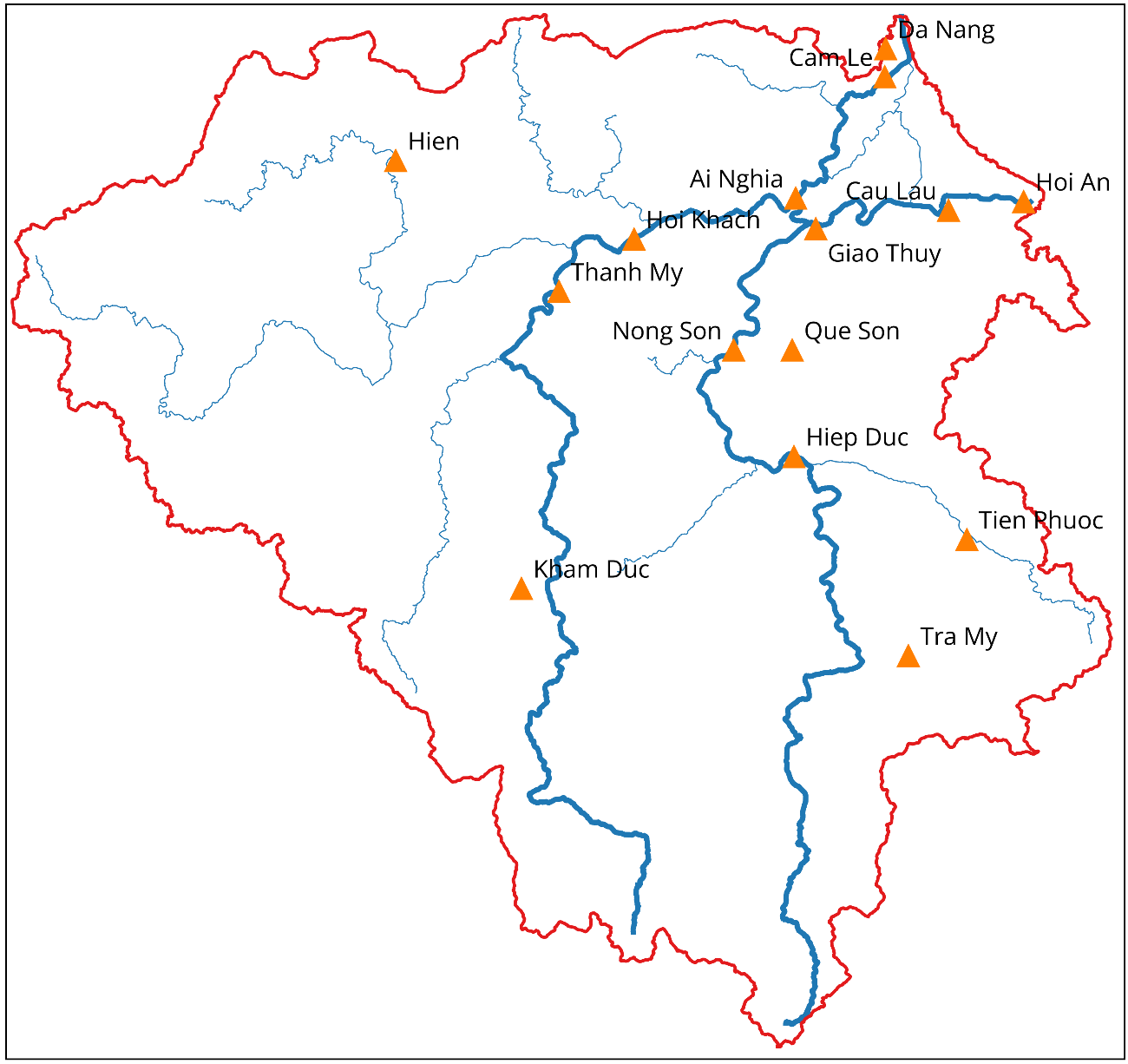


**Figure S2.** The location of 15 rain gauge stations in the VGTB basin. Maps created in the QGIS version 3.28.4 (<http://qgis.org/>) softwares^21^.


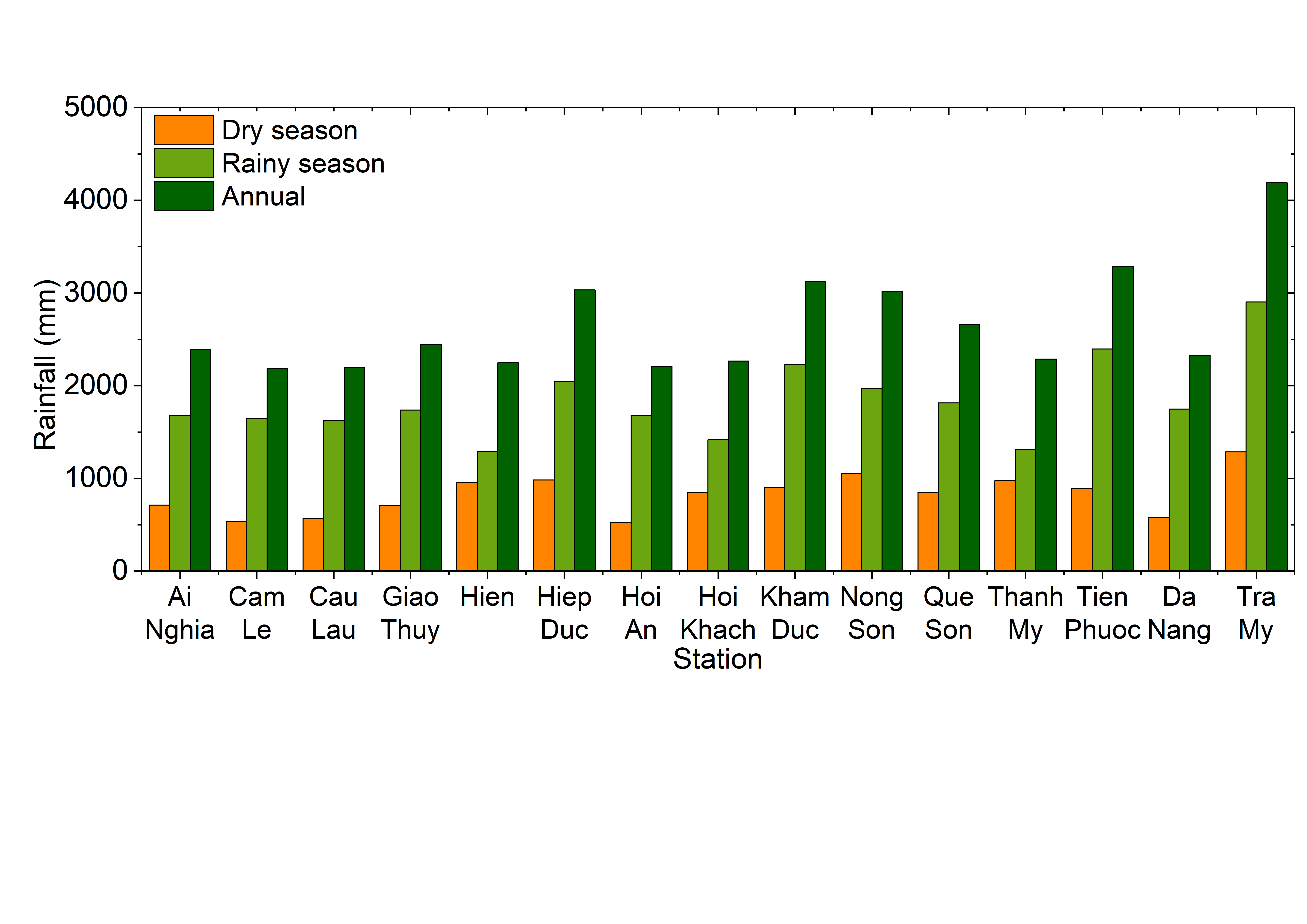


**Figure S3.** The seasonal and annual rainfall of 15 rain gauge stations in the VGTB basin.

**Table S1.** Description of SWAT datasets.

| **Data** | **Description** | **Date/ Period** | **Data sources** |
| --- | --- | --- | --- |
| Digital elevation model (DEM) | Terrain elevation, Raster, 30m x 30m | 2014 | LUCCi |
| Land use map | Land use classification, Raster, 30m x 30m | 2013 | LUCCi |
| Soil map | Soil type classification, Raster, 30m x 30m | 2011 | LUCCi |
| Meteorological data | Observed daily rainfall data at fifteen stations  Daily maximum and minimum temperature at Da Nang, Tra My, and Tam Ky stations | 1990–2020 | Hydro-meteorological station of the Central region, Vietnam |
| Streamflow, sediment data | Observed daily streamflow and sediment at Thanh My and Nong Son stations | 1990–2020 | Hydro-meteorological station of the Central region, Vietnam  Natural Disaster Prevention and Control of Quang Nam province, Vietnam |
| Dam characteristics and operation rules |  |  | Government of Vietnam, and Natural Disaster Prevention and Control of Quang Nam province |
